# Impact of BMI on perioperative outcomes following total thyroidectomy: a systematic review and meta-analysis

**DOI:** 10.1186/s13044-026-00318-2

**Published:** 2026-10-14

**Authors:** Osamah Al-Adilee, Emmanuella Habib, Dimitrios Prassas, Jasmin Mirheli, Wolfram Trudo Knoefel, Sascha Vaghiri, Levent Dizdar

**Affiliations:** 1 Department of Surgery (A), Medical Faculty, Heinrich-Heine-University, University Hospital Duesseldorf, Moorenstr. 5, 40225 Duesseldorf, Germany; 2 Medical Research School Duesseldorf, Heinrich-Heine-University Duesseldorf, Moorenstr. 5, 40225 Duesseldorf, Germany; 3 Department of Surgery, Katholisches Klinikum Essen, Philippusstift, Teaching Hospital of Duisburg-Essen University, Huelsmannstr, 17, 45355 Essen, Germany

**Keywords:** Thyroidectomy, Obesity, BMI

## Abstract

**Background:**

The influence of body mass index (BMI) on perioperative outcomes following total thyroidectomy (TT) remains controversial. We performed a systematic review and meta-analysis to evaluate the association between BMI and perioperative complications after TT.

**Methods:**

Twelve observational studies including 22,145 patients undergoing elective TT were analyzed. BMI was categorized per WHO criteria. Primary outcomes were recurrent laryngeal nerve (RLN) palsy and hypoparathyroidism. Secondary outcomes included operative time, length of stay (LOS), postoperative bleeding, and surgical site infection (SSI). Random-effects models were used.

**Results:**

BMI was not associated with RLN palsy. Lower BMI was associated with higher rates of postoperative and transient hypoparathyroidism, while permanent hypoparathyroidism did not differ. Higher BMI was associated with longer operative time and increased postoperative bleeding. LOS and SSI were comparable between groups.

**Conclusions:**

Total thyroidectomy appears to be safe in patients with elevated BMI, as no significant association was observed between BMI and the predefined primary outcomes of recurrent laryngeal nerve palsy or permanent hypoparathyroidism. Interestingly, patients with lower BMI experienced higher rates of postoperative and transient hypoparathyroidism. Higher BMI was associated with longer operative time and may be associated with an increased risk of postoperative bleeding. Therefore, particular attention should be paid to meticulous intraoperative hemostasis in patients with elevated BMI.

**Supplementary Information:**

The online version contains supplementary material available at https://doi.org/10.1186/s13044-026-00318-2.

## Introduction

Thyroidectomy is a commonly performed procedure indicated for a wide spectrum of thyroid pathologies, including thyroid cancer, goiter, and Graves’ disease. It is regarded as a safe operation, associated with low mortality rates. However, thyroidectomy carries specific risks of morbidity, such as recurrent laryngeal nerve palsy, transient or permanent hypocalcemia, and early postoperative hematoma formation [1–3]. Overweight and obesity represent a major and continuously increasing global health challenge, as emphasized by the World Health Organization (WHO). It is strongly associated with a wide range of comorbidities, including type 2 diabetes mellitus, cardiovascular diseases, and several malignancies [4–6].

A large body of evidence has demonstrated a robust association between elevated body mass index (BMI) and increased morbidity and mortality, particularly in the context of surgical procedures and perioperative outcomes [7–13].

Numerous studies have examined the relationship between body mass index (BMI) and postoperative complications following thyroidectomy; however, their findings remain inconsistent and often contradictory. For instance, Buerba et al. demonstrated an increased incidence of overall and wound-related complications among patients with obesity undergoing thyroid surgery. In contrast, Blanchard et al. and Vaghiri et al. found no statistically significant differences in complication rates between patients with obesity and patients without obesity, although both observed prolonged operative times inindividuals with obesity. Conversely, Soelling et al. reported that a higher BMI appeared to be protective against postoperative hypocalcemia [14–17]. These inconsistencies in the published literature are most likely attributable to insufficient statistical power, driven by small sample sizes and the low incidence of key complications such as RLN injury and hypoparathyroidism. By pooling data across individual studies, this meta-analysis aims to provide a more robust evaluation of the potential impact of overweight and obesity on surgical outcomes following thyroidectomy. A clearer understanding of these relationships may support the development of targeted strategies to mitigate complication risks in the growing population of patients with overweight or obesity.

## Materials and methods

This meta-analysis was conducted in accordance with the Preferred Reporting Items for Systematic Reviews and Meta-Analyses (PRISMA) statements and Cochrane Handbook for Systematic Reviews and of Interventions [18, 19]. The study protocol was registered in the International Prospective Register of Systematic Reviews (PROSPERO; registration number: CRD420251238418).

### Study aim and PICO(S) framework

This meta-analysis sought to determine the impact of Body Mass Index (BMI) on the incidence of postoperative complications following total thyroidectomy (TT). The study elements were defined according to the PICO(S) framework [20]:

- P – Population: Adult patients (≥ 18 years old) undergoing elective conventional open TT with or without neck dissection for any indication (including benign and malignant thyroid disease).
- I – Intervention/Exposure: TT in patients without overweight (BMI < 25 kg/m²) and patients without obesity (BMI < 30 kg/m²), as defined by the World Health Organization (WHO) [5].
- C – Comparison: TT In patients with overweight (BMI ≥ 25 kg/m²) and patients with obesity (BMI ≥ 30 kg/m²).
- O – Outcomes:
  - Primary Outcomes: Incidence of Recurrent Laryngeal Nerve (RLN) palsy and postoperative hypoparathyroidism.
  - Secondary Outcomes: Surgical Site Infection (SSI), length of hospital stay (LOS), postoperative bleeding and hematoma, and operating room (OR) time.
- S – Study Design: Observational studies (prospective or retrospective cohort studies), including both single-centre or multi-centre designs.

### Search strategy

A comprehensive systematic search was conducted in PubMed/MEDLINE, the Cochrane Central Register of Controlled Trials (CENTRAL), and Google Scholar. The search strategy was developed using a combination of Medical Subject Headings (MeSH) terms and free-text keywords, applying the terms BMI, obesity, and thyroidectomy. No restrictions on publication date or language were applied. Duplicate records were identified and removed. Two reviewers (S.V., L.D.) independently screened the titles and abstracts of the remaining records against predefined eligibility criteria. Potentially relevant articles were retrieved in full text, and their reference lists were manually screened to identify additional studies (snowballing). The same two reviewers independently assessed the full texts for final eligibility. Any discrepancies at either stage were resolved through discussion and consensus. The last search was performed on April 2025.

### Eligibility criteria

We included full-text articles published in international peer-reviewed international journals that reported on adult patients undergoing conventional open total thyroidectomy (TT) and provided comparative data stratified by BMI categories. Studies were excluded if they involved patients undergoing partial thyroidectomy (e.g., lobectomy, hemithyroidectomy, subtotal thyroidectomy), alternative surgical approaches (e.g., endoscopic, robotic, transoral, transaxillary), parathyroidectomy, or other concomitant cervical procedures. We also excluded studies in which BMI stratification was not clearly reported or where data could not be synthesized for pooled analysis.

### Data extraction

Data from all included studies were independently extracted by three authors [O.A., E.H., and J.M.] using a standardized, pre-piloted data extraction form in Microsoft Excel. Discrepancies were resolved through discussion between the reviewers or by consultation with a third reviewer [S.V. and L.D.]. We extracted the following key information from each study:

- Study characteristics: First author, year and country of publication, study period and study design, total number of participants, underlying thyroid disease (benign/malignant), if central or lateral lymph node dissection (LND) was performed, duration of postoperative follow-up, study endpoints, BMI definitions and cut-off values.
- Patient demographics and baseline characteristics: Mean age, mean BMI, sex distribution, nodule size, use of energy devices (e.g., LigaSure™, Harmonic™), application of intraoperative neuromonitoring. Information on parathyroid auto transplantation and placement of surgical drains (if reported).
- Surgical data and clinical outcomes: Operating room (OR) time (minutes), length of hospital stay (LOS) in days, incidence of surgical site infection (SSI), postoperative bleeding and hematoma, incidence of postoperative Hypoparathyroidism (HPT), recurrent laryngeal nerve (RLN) palsy, and length of follow-up period.

### Definition of primary outcomes

- Recurrent laryngeal nerve (RLN) palsy: Definitions of RLN palsy varied across studies. Reported terms included abnormal vocal cord motility, vocal cord paralysis, vocal cord dysfunction, and hoarseness. In most studies, postoperative laryngoscopy was performed. Many studies further differentiated between transient and permanent dysfunction, with permanent complications defined as dysfunction persisting for more than 6 or 12 months. For our analysis, we categorized RLN palsy into three categories: (1) Postoperative RLN palsy (all postoperative dysfunctions - transient and permanent). (2) Transient RLN palsy. (3) Permanent RLN palsy.
- Postoperative Hypoparathyroidism: The definition of postoperative hypoparathyroidism also varied among the included studies. Reduced calcium or parathyroid hormone levels below the normal range were used as diagnostic criteria. Similar to RLN palsy, several studies distinguished between transient and permanent hypoparathyroidism, with the permanent form defined as dysfunction persisting for more than 6 or 12 months. Consequently, we applied the same categorization approach as described above, dividing postoperative hypoparathyroidism into: (1) postoperative hypoparathyroidism (transient and permanent), (2) transient hypoparathyroidism, and (3) permanent hypoparathyroidism.

### Risk of bias and certainty of evidence assessment

The risk of bias of the included studies was independently assessed by two reviewers [O.A., E.H.] using the Risk Of Bias In Non-randomized Studies - of Interventions Version 2 (ROBINS-I V2) tool (2). The ROBINS-I tool evaluates bias across seven domains: confounding, participant selection, classification of interventions, deviations from intended interventions, missing data, outcomes measurement, and selective reporting. Each domain is rated as low, moderate, serious, or critical risk of bias. The reviewers were not blinded to the study authors. Any disagreements in the risk of bias assessment were resolved by consulting a third reviewer [S.V.]. The Grading of Recommendations, Assessment, Development, and Evaluation (GRADE) methodology was used to adequately document the strength of evidence for the main significant outcomes, implementing four levels of evidence (high, moderate, low, and very low) [21, 22].

### Statistical analysis

All statistical analyses were performed using Review Manager (RevMan) software (version 5.4. Copenhagen: The Nordic Cochrane Centre, The Cochrane Collaboration, 2014) according to the recommendations of the Cochrane Collaboration guidelines. For dichotomous outcomes, pooled Odds Ratios (ORs) with 95% Confidence Intervals (CIs) and the Mantel-Haenszel weights were calculated. For continuous outcomes, Standardized Mean Differences (SMDs) with 95% CIs were calculated. The effect size of continuous outcomes was summarized using the inverse-variance method. To estimate the sample, mean and standard deviation from studies reporting medians, interquartile ranges, and sample sizes, we applied the methods proposed by Luo et al. [23] and Wan et al. [24], as well as the Box-Cox (BC) method described by McGrath et al. [25].

Heterogeneity among studies was assessed using the I^2^ statistic and Cochran’s Q test (chi-squared test). An I^2^ value of < 25% was considered low heterogeneity, 25 − 50% moderate, and > 50% substantial heterogeneity. When heterogeneity was low to moderate (I² < 50%), summary estimates were calculated using a fixed-effects model. If heterogeneity exceeded this threshold (I² > 50%), a random-effects model was applied. In cases of substantial heterogeneity, potential sources were further explored using leave-one-out sensitivity analysis [19].

The overall effect for each outcome, and for each subgroup, was determined by calculating a Z-statistic, with statistical significance set at a two-sided p-value of < 0.05.

## Results

**Fig. 1:**
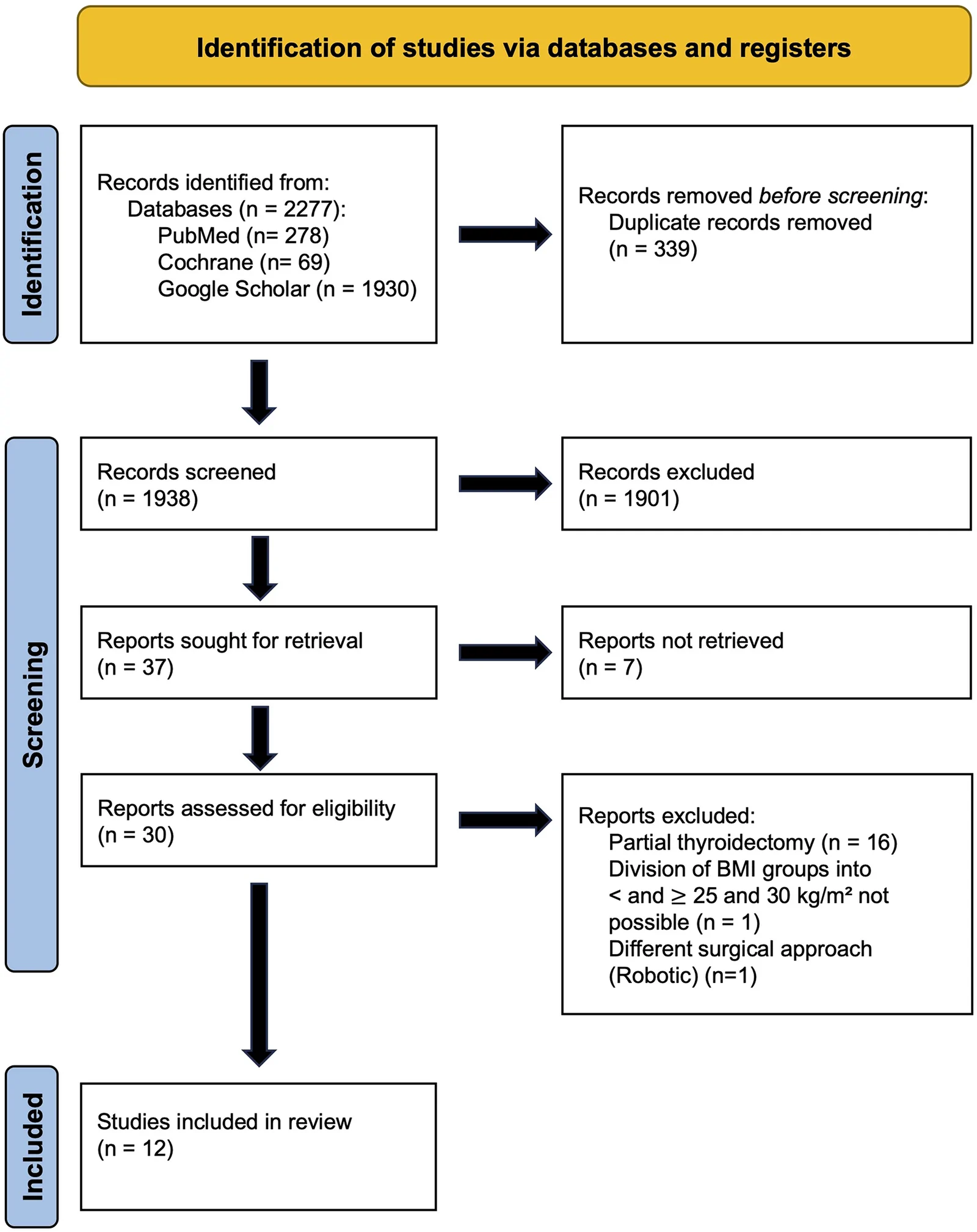
PRISMA diagram of study identification and selection for meta-analysis

The study selection and inclusion process is summarized in the PRISMA flow diagram (Fig. 1). Of 2 277 articles initially identified through electronic databases, 12 studies ultimately met the inclusion criteria and were eligible for the final meta-analysis [14–16, 26–34].

### Study and patient characteristics

The meta-analysis included 12 studies, encompassing a total of 22 145 patients from 5 different countries, with study periods ranging from 2002 to 2022.

Regarding study design, two were prospective cohort studies [15, 31], five were retrospective cohort studies [28–30, 32, 33], and five were database analyses [14, 16, 26, 27, 34]. Ten studies were single-center investigations, while two were multi-center. Reported follow-up periods ranged from 30 days to 87 months. A detailed summary of the included studies’ characteristics, including their publication year, country of origin, specific study design, sample size, performance of lymph node dissection (LND), follow-up duration, and original BMI definitions used, is provided in Table 1.

All studies included patients undergoing thyroidectomy for both malignant and benign thyroid disease, except for three studies that focused exclusively on malignant cases [32, 33] and one study that did not specify the indication [26].

For our comparative analyses, patient demographic and clinical characteristics were assessed across two main BMI thresholds: patients with BMI ≥ 25 kg/m^2^ versus those with BMI < 25 kg/m^2^, and patients with BMI ≥ 30 kg/m^2^ versus those with BMI < 30 kg/m^2^. For each of these comparison frameworks, detailed characteristics such as the number of patients, mean age, gender distribution, use of intraoperative neuromonitoring, and additional data, are provided in Table 2 (for BMI ≥ 25 vs. < 25 kg/m^2^) and Table 3 (for BMI ≥ 30 vs. < 30 kg/m^2^).

**Table 1:** Study characteristics

Author	Year	Origin	Study period	Study design	Sample size (*n*)	Patients analyzed (*n*)	Thyroid disease (*n*)	Follow-up period	Endpoints
Blanchard et al. [18]	2019	France	2012–2014	Prospective, randomized, multi-center, single blind study	1310	1310	Benign (1025), malignant (285)	6 months	Primary: hypocalcemia, RLN palsy, hematoma Secondary: operative time, postoperative pain, LOS
Canu et al. [33]	2020	Italy	2014–2018	Retrospective cohort study, single center	1594	813	Benign (503), malignant (307)	12 months	Primary: postoperative complications Secondary: operative time, postoperative stay
Celik et al. [37]	2021	Turkey	2014–2017	Retrospective review prospective database, Single center	161	144	Benign (65), malignant (79)	12 months	Body composition parameters and hypocalcemia
Eminov et al. [34]	2015	Turkey	2012	Prospective, observational study, Single center	99	99	Benign (74), malignant (25)	NR	Incision length, operative time, specimen weight, pain, early complications, drainage, LOS
Feng et al. [36]	2019	China	2012–2018	Retrospective Study, Single center	487	417	Malignant	5–87 months	Effects of excess body weight on clinicopathologic features of PTC
Mahvi et al. [32]	2022	USA	2016–2019	Single-center retrospective study	559	559	Benign (157), malignant (314)	30 days	Postoperative hypocalcemia
Milone et al. [31]	2016	Italy	2007–2013	Retrospective cohort study, Single center	266	266	Benign (192), malignant (74)	6 months	Primary: hypocalcemia, RLN palsy, and hematoma Secondary: operative time, LOS
Özdemir et al. [30]	2022	Turkey	2009–2016	Retrospective review, Single center	601	415	Benign (409), malignant (6)	1 year	LOS, operative time, postoperative histopathology, complications (hypocalcemia, RLN injury, hematoma, seroma, wound infection)
Soelling et al. [17]	2023	USA	2016–2020	Retrospective analysis, Multicenter	23,899	16,277	Benign (12745), malignant (3125)	30 days	Hypocalcemia
Tresallet et al. [35]	2014	France	2002–2009	Retrospective Study, Single center	6684	1216	Malignant	7 years	Incidence of papillary thyroid carcinoma and outcomes in operative patients according to their body mass indices
Üstün et al. [29]	2020	Turkey	2015–2018	Retrospective review, Single center	145	145	NR	6 months	Operative time, postoperative complications (SSI, bleeding, hypocalcemia, RLN injury)
Vaghiri et al. [19]	2025	Germany	2010–2022	Retrospective review of prospectively collected data, single center	498	484	Benign (242), malignant (49)	6 months	Postoperative RLN palsy and hypoparathyroidism

RLN: Recurrent laryngeal nerve; SSI: Surgical site infection; LOS: Length of stay; NR: Not reported; BMI: Body mass index;

**Table 2:** Characteristics of patients with BMI < 25 kg/m² vs. ≥ 25 kg/m²

Author	Group	Number of patients (*n*)	Age (mean/SD)	BMI (mean/SD)	Gender (M/F)	Nodule size (mean/SD)	Use of energy devices (*n*)	Neuromonitoring RLN (*n*)	Parathyroid Transplantation (*n*)
Blanchard et al. [18]	BMI < 25	595	48.6 ± 13.5	NR	74/521	NR	NR	NR	NR
	BMI ≥ 25	715	52.5 ± 13.1	NR	187/528	NR	NR	NR	NR
Eminov et al. [34]	BMI < 25	25	46.6 ± 15.5	NR	8/17	NR	25	NR	NR
	BMI ≥ 25	74	51.7 ± 12	NR	20/54	NR	74	NR	NR
Feng et al. [36]	BMI < 25	260	43.9 ± 12.2	NR	51/209	1.19 ± 0.93	NR	NR	NR
	BMI ≥ 25	157	48.2 ± 11.2	NR	55/102	1.26 ± 0.93	NR	NR	NR
Mahvi et al. [32]	BMI < 25	168	46.5 ± 16.1	NR	29/139	NR	NR	NR	30
	BMI ≥ 25	390	51.6 ± 14.8	NR	86/304	NR	NR	NR	75
Milone et al. [31]	BMI < 25	104	43 ± 10.6	NR	65/39	77.6 ± 44.3	No	No	NR
	BMI ≥ 25	162	40.9 ± 11.1	NR	88/74	83.9 ± 35.4	No	No	NR
Özdemir et al. [30]	BMI < 25	81	NA	23.1 ± 1.3	3/78	NR	NR	NR	NR
	BMI ≥ 25	334	NA	30.6 ± 2.8	97/237	NR	NR	NR	NR
Soelling et al. [17]	BMI < 25	3531	NA	NR	576/2955	NR	2182	2181	412
	BMI ≥ 25	12,595	NA	NR	3084/9511	NR	8435	8509	1340
Tresallet et al. [35]	BMI < 25	684	48 ± 15	NR	104/580	13.4 ± 12.5	NR	NR	NR
	BMI ≥ 25	532	52.7 ± 12.7	NR	157/375	13.4 ± 13.7	NR	NR	NR
Üstün et al. [29]	BMI < 25	66	43.9 ± 0	NR	5/61	NR	NR	NR	1
	BMI ≥ 25	79	48.3 ± 0	NR	15/64	NR	NR	NR	7

SD: Standard deviation; BMI: Body mass index; M: Male; F: Female; RLN: Recurrent laryngeal nerve; NR: Not reported;

**Table 3:** Characteristics of patients with BMI < 30 kg/m² vs. ≥ 30 kg/m²

Author	Group	Number of patients (*n*)	Age (mean/SD)	BMI (mean/SD)	Gender (M/F)	Nodule size (mean/SD)	Use of energy devices (*n*)	Neuromonitoring RLN (*n*)	Parathyroid Transplantation (*n*)
Blanchard et al. [18]	BMI < 30	1020	50.4 ± 13.6	NR	197/ 823	NR	NR	635	NR
	BMI ≥ 30	290	52.0 ± 12.7	NR	64/ 226	NR	NR	165	NR
Canu et al. [33]	BMI < 30	678	54.0 ± 14.1	24.3 ± 3.3	198/480	NR	678	678	NR
	BMI ≥ 30	135	56.2 ± 14	34 ± 4	40/95	NR	135	135	NR
Celik et al. [37]	BMI < 30	66	NR	NR	NA	NR	NR	NR	NR
	BMI ≥ 30	78	NR	NR	NA	NR	NR	NR	NR
Eminov et al. [34]	BMI < 30	61	50.3 ± 14	NR	19/42	NR	61	NR	NR
	BMI ≥ 30	38	50.7 ± 11.3	NR	9/29	NR	38	NR	NR
Feng et al. [36]	BMI < 30	392	43.5 ± 16.2	NR	89/303	1.21 ± 0.94	NR	NR	NR
	BMI ≥ 30	25	49 ± 8	NR	17/8	1.32 ± 0.76	NR	NR	NR
Özdemir et al. [30]	BMI < 30	245	NR	26.0 ± 2.3	89/156	NR	NR	NR	NR
	BMI ≥ 30	170	NR	33.7 ± 1.3	11/159	NR	NR	NR	NR
Soelling et al. [17]	BMI < 30	8354	NR	NR	1858/6496	NR	5285	5293	917
	BMI ≥ 30	7772	NR	NR	1802/5970	NR	5332	5397	835
Tresallet et al. [35]	BMI < 30	1040	49.7 ± 14.5	NR	215/825	13.4 ± 13	NR	NR	NR
	BMI ≥ 30	176	52 ± 12	NR	46/130	13.1 ± 13.1	NR	NR	NR
Vaghiri et al. [19]	BMI < 30	193	52.2 ± 10.8	24.3 ± 3.3	67/126	NR	NR	NR	NR
	BMI ≥ 30	98	51.5 ± 11.6	34.4 ± 4.2	34/64	NR	NR	NR	NR

SD: Standard deviation; BMI: Body mass index; M: Male; F: Female; RLN: Recurrent laryngeal nerve; NR: Not reported;

### Study quality and risk of bias

**Fig. 2:**
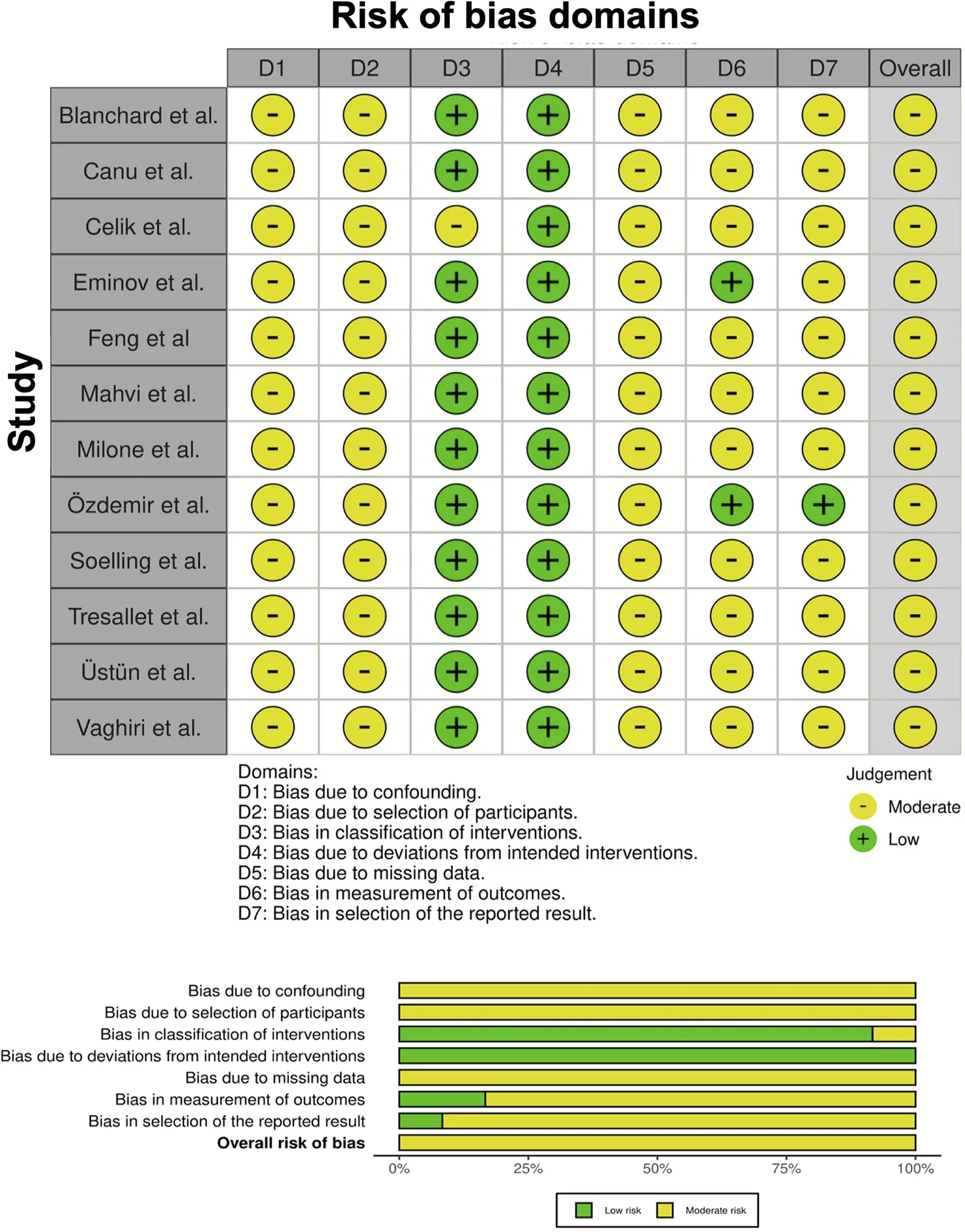
Graphical summary of risk of bias in the included studies based on ROBINS-I tool

Study quality and risk of bias were evaluated using the Risk of Bias in Non-Randomized Studies of Interventions (ROBINS-I) tool. All included studies demonstrated a moderate risk of bias (Fig. 2). The primary sources of bias were related to the inherent limitations of the non-randomized study design and inconsistencies in outcome definitions.

### Primary outcome analysis

The primary outcomes of the included studies were recurrent laryngeal nerve palsy and hypoparathyroidism. The results are presented in Tables S2 and S3.

#### Recurrent laryngeal nerve palsy

##### BMI < 25 kg/m^2^ versus BMI ≥ 25 kg/m^2^

In the comparison between patients with BMI < 25 kg/m^2^ and ≥ 25 kg/m^2^, postoperative recurrent laryngeal nerve (RLN) events were reported in eight studies [14, 15, 26–28, 31–33] comprising a total of 19 994 patients. Transient RLN palsy was reported in five [15, 26–28, 32] studies including 3 352 patients, and permanent RLN palsy was reported in the same five [15, 26–28, 32] studies involving the same patient cohort.

**Fig. 3:**
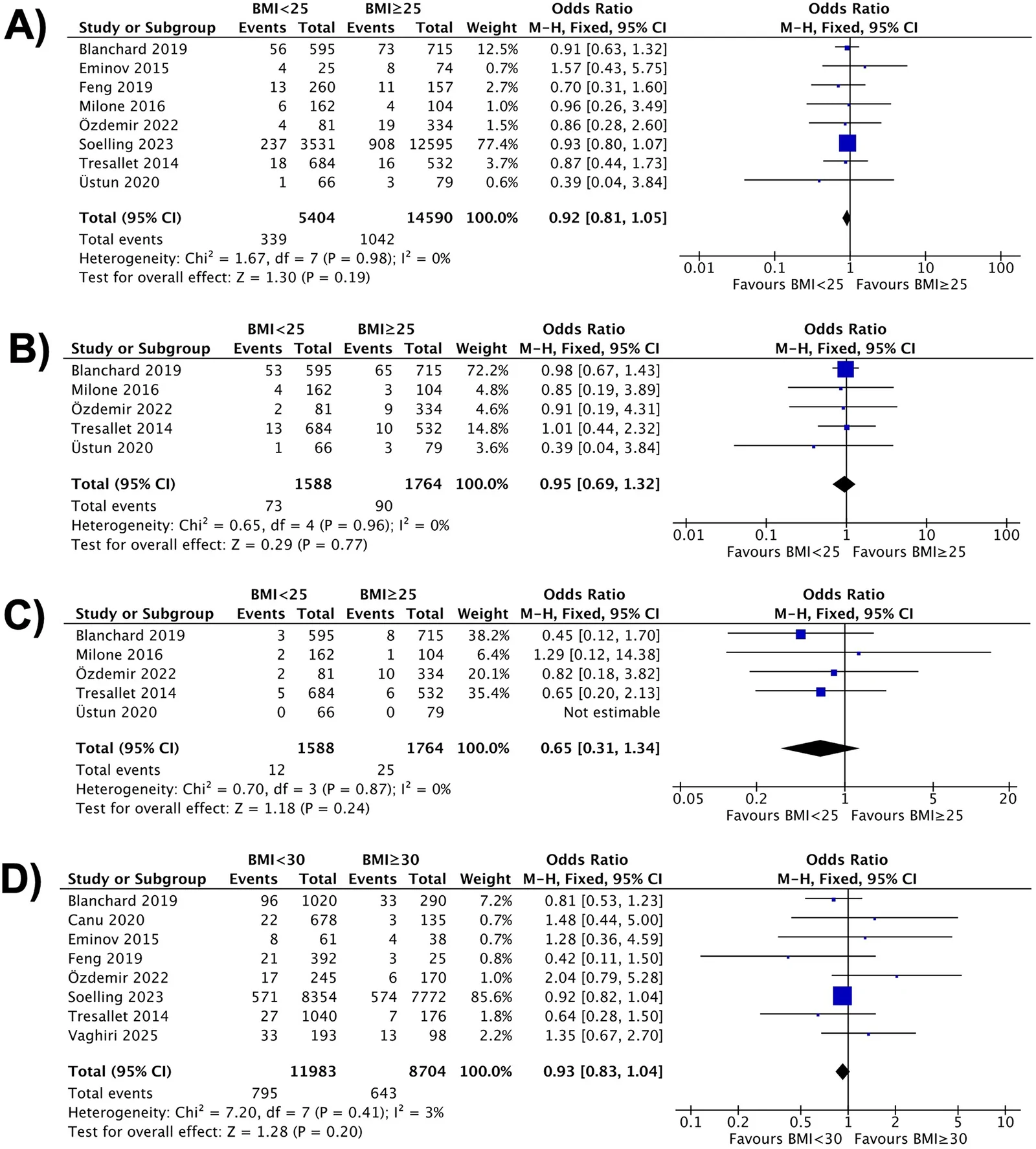

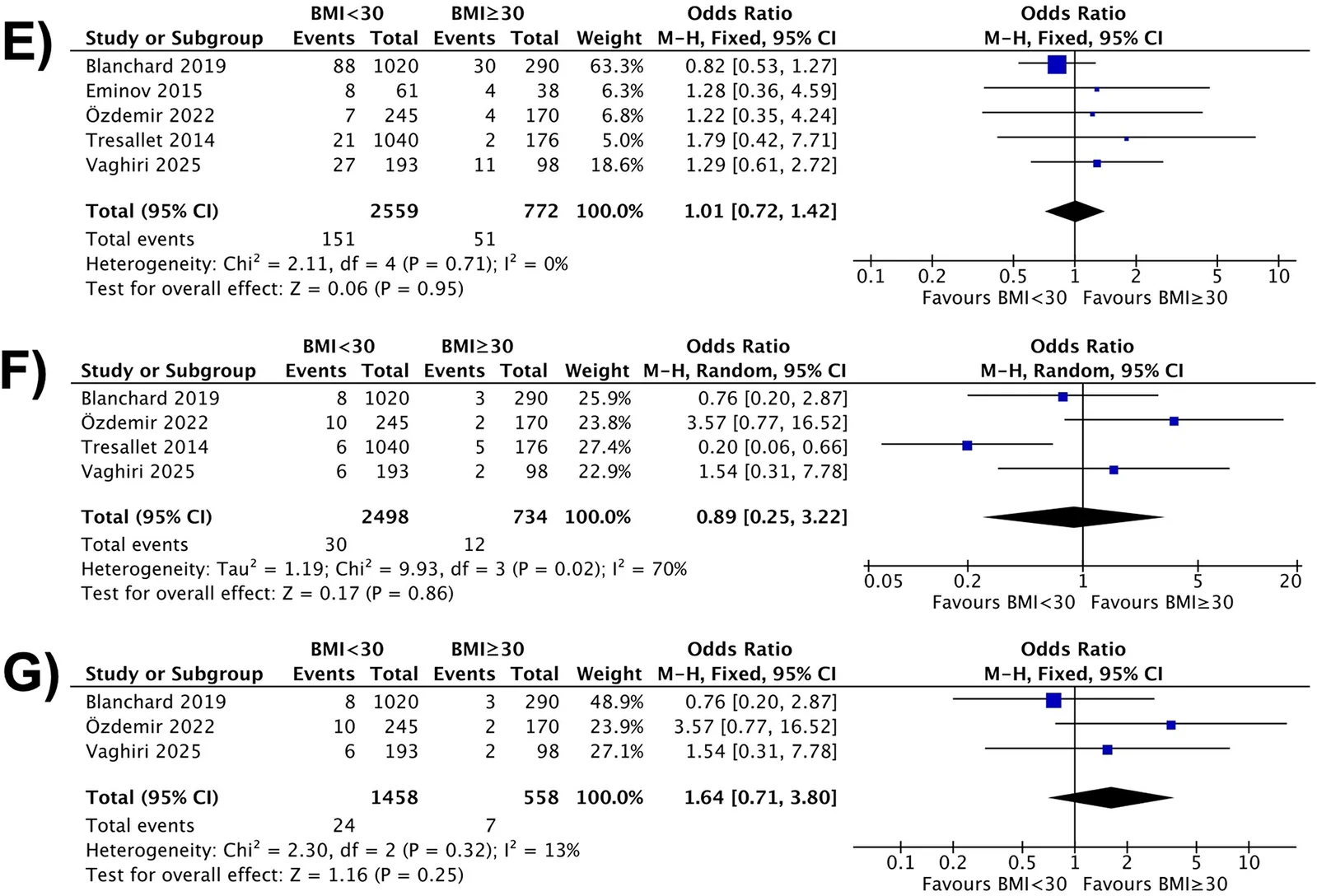
(**A**) Forest plot comparing the postoperative recurrent laryngeal nerve palsy in two BMI groups of BMI < 25 kg/m^2^ and ≥ 25 kg/m^2^. (**B**) Forest plot comparing the transient recurrent laryngeal nerve palsy in two BMI groups of BMI < 25 kg/m^2^ and ≥ 25 kg/m^2^. (**C**) Forest plot comparing the permanent recurrent laryngeal nerve palsy in two BMI groups of BMI < 25 kg/m^2^ and ≥ 25 kg/m^2^. (**D**) Forest plot comparing the postoperative recurrent laryngeal nerve palsy in two BMI groups of BMI < 30 kg/m^2^ and ≥ 30 kg/m^2^. (**E**) Forest plot comparing the transient recurrent laryngeal nerve palsy in two BMI groups of BMI < 30 kg/m^2^ and ≥ 30 kg/m^2^. (**F**) Forest plot comparing the permanent recurrent laryngeal nerve palsy in two BMI groups of BMI < 30 kg/m2 and ≥ 30 kg/m^2^. (**G**) Leave-one-out sensitivity analysis: Forest plot comparing permanent recurrent laryngeal nerve (RLN) palsy between patients with BMI < 30 kg/m^2^ and ≥ 30 kg/m^2^

Statistical analysis of overall postoperative RLN palsy showed no significant difference between the two BMI groups (OR 0.92; 95% CI: 0.81–1.05; *p* = 0.19). Heterogeneity among the studies was low (I² = 0%; Chi² test: *p* = 0.98) (Fig. 3A).

Similarly, no significant difference was observed in the incidence of transient RLN palsy between the two BMI groups (OR 0.95; 95% CI: 0.69–1.32; *p* = 0.77). Heterogeneity among the studies was low (I² = 0%; Chi² test: *p* = 0.96) (Fig. 3B). For permanent RLN palsy, no statistically significant difference was found (OR 0.65; 95% CI: 0.31–1.34; *p* = 0.24), and heterogeneity remained low (I² = 0%; Chi² test: *p* = 0.87) (Fig. 3C).

##### BMI < 30 kg/m^2^ versus BMI ≥ 30 kg/m^2^

In the analysis comparing patients with BMI < 30 kg/m^2^ and ≥ 30 kg/m^2^, postoperative recurrent laryngeal nerve (RLN) events were reported in eight [14–16, 27, 30–33] studies comprising a total of 20 687 patients. Transient and permanent RLN palsy were reported in five [15, 16, 27, 31, 32] studies including 3 331 and 3 232 patients, respectively.

Statistical analysis of overall postoperative RLN palsy showed no significant difference between the two BMI groups (OR 0.93; 95% CI: 0.83–1.04; *p* = 0.20). Heterogeneity among the studies was low (I² = 3%; Chi² test: *p* = 0.41) (Fig. 3D).

Similarly, no significant difference was observed in the incidence of transient RLN palsy between the two BMI groups (OR 1.01; 95% CI: 0.72–1.42; *p* = 0.95). Heterogeneity among the studies was low (I² = 0%; Chi² test: *p* = 0.71) (Fig. 3E). For permanent RLN palsy, no statistically significant difference was found (OR 0.89; 95% CI: 0.25–3.22; *p* = 0.86), and heterogeneity was substantial (I² = 70%; Chi² test: *p* = 0.02) (Fig. 3F). Due to the substantial heterogeneity, a leave-one-out sensitivity analysis was performed. This analysis identified the study by Tresallet et al. [32] as the primary source of heterogeneity. After excluding this study, heterogeneity markedly decreased (I² = 13%; Chi² test: *p* = 0.32) and the pooled results were recalculated using a fixed-effects model, confirming the robustness of the findings (Fig. 3G).

#### Hypoparathyroidism

##### BMI < 25 kg/m^2^ versus BMI ≥ 25 kg/m^2^

**Fig. 4:**
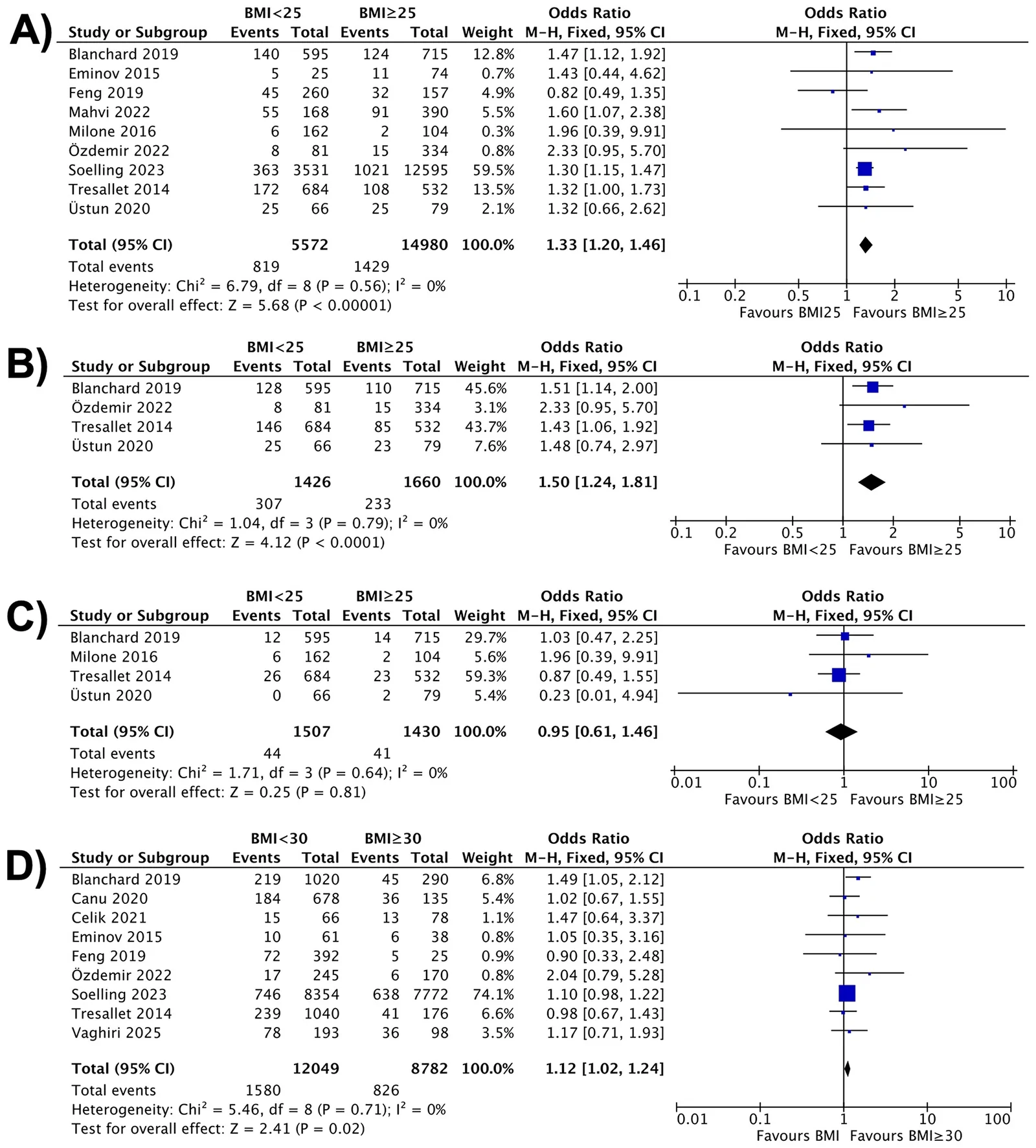

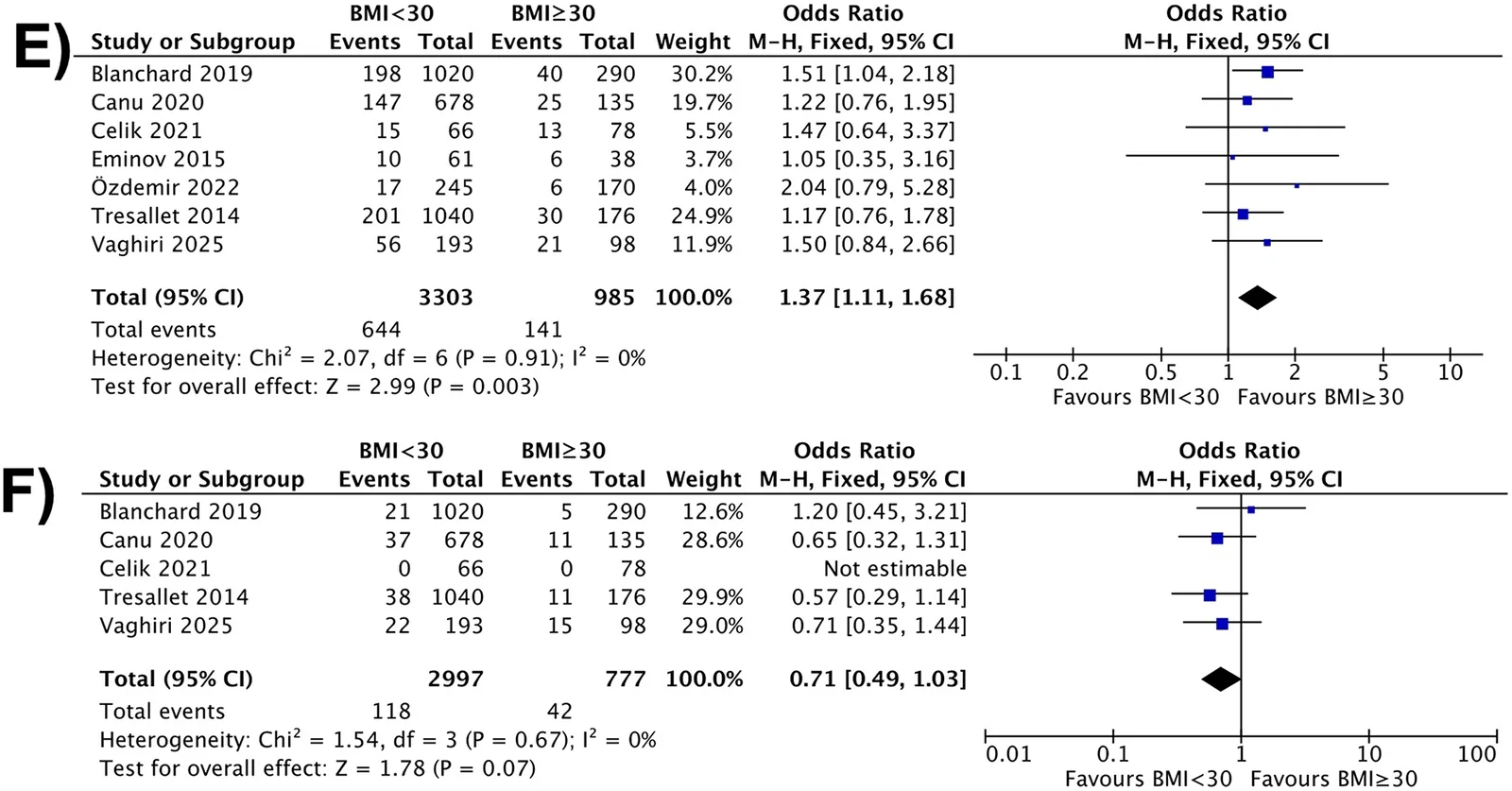
(**A**) Forest plot comparing the postoperative hypoparathyroidism in two BMI groups of BMI < 25 kg/m^2^ and ≥ 25 kg/m^2^. (**B**) Forest plot comparing the transient hypoparathyroidism in two BMI groups of BMI < 25 kg/m^2^ and ≥ 25 kg/m^2^. (**C**) Forest plot comparing the permanent hypoparathyroidism in two BMI groups of BMI < 25 kg/m^2^ and ≥ 25 kg/m^2^. (**D**) Forest plot comparing the postoperative hypoparathyroidism in two BMI groups of BMI < 30 kg/m^2^ and ≥ 30 kg/m^2^. (**E**) Forest plot comparing the transient hypoparathyroidism in two BMI groups of BMI < 30 kg/m2 and ≥ 30 kg/m^2^. (**F**) Forest plot comparing the permanent hypoparathyroidism in two BMI groups of BMI < 30 kg/m^2^ and ≥ 30 kg/m^2^

Our analysis of nine studies [14, 15, 26–29, 31–33] comprising a total of 20 552 patients showed that postoperative hypoparathyroidism occurred significantly more frequently in patients with a BMI < 25 kg/m^2^ compared with those with a BMI ≥ 25 kg/m^2^ (OR 1.33; 95% CI: 1.20–1.46; *p* < 0.00001) (Fig. 4A). The level of heterogeneity was low (I² = 0%; Chi² test: *p* = 0.56) and the level of certainty of evidence based on the GRADE criteria was low (Tab. S6).

Transient hypoparathyroidism was also reported significantly more often in the lower BMI group in four [15, 26, 27, 32] studies including 3 086 patients (OR 1.50; 95% CI: 1.24–1.81; *p* < 0.0001) (Fig. 4B). The level of heterogeneity was low (I² = 0%; Chi² test: *p* = 0.79). The quality of evidence was low according to the GRADE criteria (Tab. S6). In contrast, no statistically significant difference was observed for permanent hypoparathyroidism, which was reported in four [15, 26, 28, 32] studies with a total of 2 937 patients (OR 0.95; 95% CI: 0.61–1.46; *p* = 0.81) (Fig. 4C). Heterogeneity was also low (I² = 0%; Chi² test: *p* = 0.64).

##### BMI < 30 kg/m^2^ versus BMI ≥ 30 kg/m^2^

In the comparison between patients with a BMI < 30 kg/m^2^ and those with a BMI ≥ 30 kg/m^2^, postoperative hypoparathyroidism occurred significantly more often in the lower BMI group across nine studies [14–16, 27, 30–34] involving a total of 20 831 patients (OR 1.12; 95% CI: 1.02–1.24; *p* = 0.02) (Fig. 4D). The level of heterogeneity was low (I² = 0%; Chi² test: *p* = 0.71) and the level of certainty of evidence based on the GRADE criteria was low (Tab. S7). Transient hypoparathyroidism was reported in seven studies [15, 16, 27, 30–32, 34] including 4 288 patients. Statistical analysis showed a significantly higher incidence of transient hypoparathyroidism in the BMI < 30 kg/m^2^ group (OR 1.37; 95% CI: 1.11–1.68; *p* = 0.003) (Fig. 4E). Heterogeneity among the studies was low (I² = 0%; Chi² test: *p* = 0.91) and the quality of evidence was low according to the GRADE criteria (Tab. S7). In contrast, analysis of five studies [15, 16, 30, 32, 34] with a total of 3 774 patients revealed no statistically significant difference in the incidence of permanent hypoparathyroidism between the two BMI groups (OR 0.71; 95% CI: 0.49–1.03; *p* = 0.07) (Fig. 4F). Heterogeneity among the studies was low (I² = 0%; Chi² test: *p* = 0.67).

### Secondary outcome analysis

The secondary outcomes assessed in the included studies were operative time, length of hospital stay, postoperative bleeding, and surgical site infection. The results are presented in Tables S4 and S5.

#### Operative time

##### BMI < 25 kg/m^2^ versus BMI ≥ 25 kg/m^2^

**Fig. 5:**
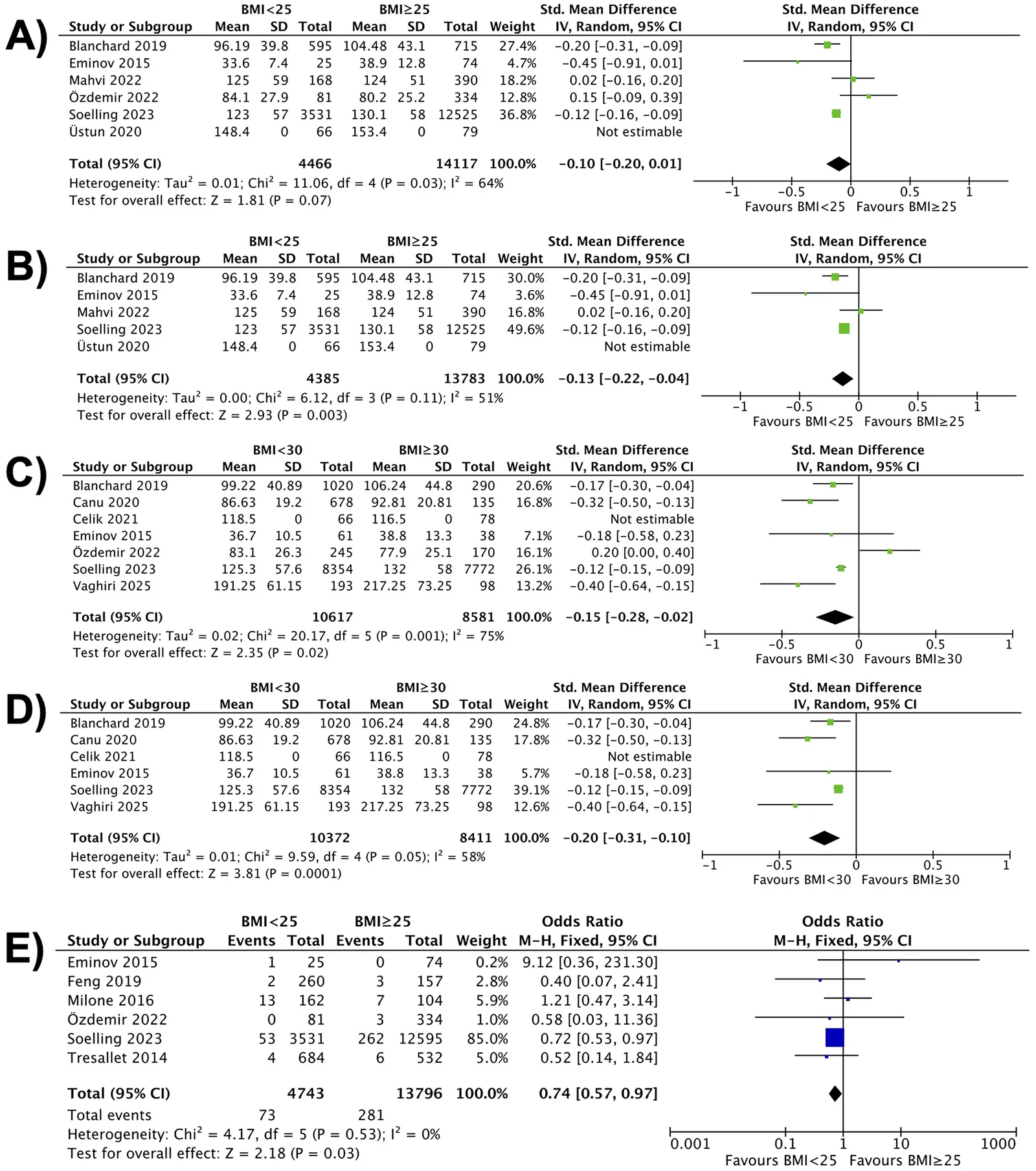

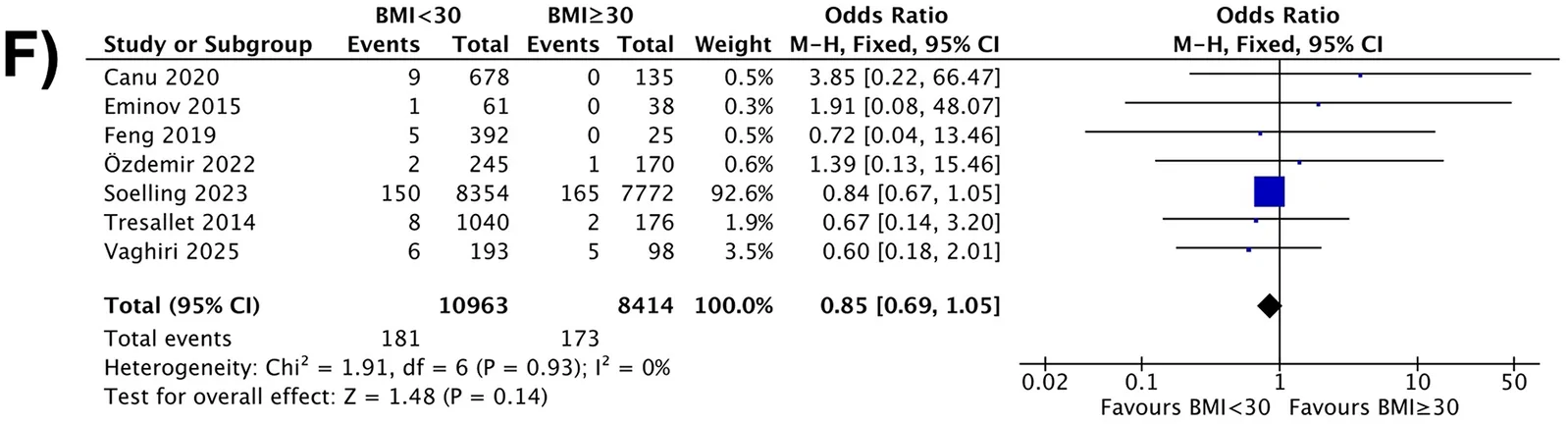
(**A**) Forest plot comparing operative time between patients with BMI < 25 kg/m^2^ and ≥ 25 kg/m^2^. (**B**) Leave-one-out sensitivity analysis: Forest plot comparing operative time between patients with BMI < 25 and ≥ 25. (**C**) Forest plot comparing operative time between patients with BMI < 30 kg/m^2^ and ≥ 30 kg/m^2^. (**D**) Leave-one-out sensitivity analysis: Forest plot comparing operative time between patients with BMI < 30 kg/m2 and ≥ 30 kg/m^2^. (**E**) Forest plot comparing postoperative bleeding between patients with BMI < 25 kg/m^2^ and ≥ 25 kg/m^2^ (**F**) Forest plot comparing postoperative bleeding between patients with BMI < 30 kg/m^2^ and ≥ 30 kg/m^2^

Operative time was reported in six studies [14, 15, 26, 27, 29, 31] including a total of 18 583 patients. One of these studies [26] did not report a standard deviation (SD), which rendered its data non-estimable for pooling in the meta-analysis. Consequently, five studies [14, 15, 27, 29, 31] were quantitatively analyzed. The initial pooled estimate suggested a small reduction in operative time in patients with BMI < 25 kg/m^2^ (SMD − 0.10; 95% CI: − 0.20 to 0.01; *p* = 0.07) (Fig. 5A). The level of heterogeneity was substantial (I² = 64%; Chi² test: *p* = 0.03). Due to this substantial heterogeneity, a leave-one-out sensitivity analysis was performed, which identified the study by Özdemir et al. ^27^ as the primary source of heterogeneity. After excluding this study, heterogeneity decreased (I² = 51%; Chi² test: *p* = 0.11) and the pooled results were recalculated using a random-effects model, demonstrating a significantly shorter operative time in patients with BMI < 25 kg/m^2^ (SMD − 0.13; 95% CI: − 0.22 to − 0.04; *p* = 0.003) (Fig. 5B). The quality of evidence was very low according to the GRADE criteria (Tab. S6).

##### BMI < 30 kg/m^2^ versus BMI ≥ 30 kg/m^2^

Operative time was reported in seven studies [14–16, 27, 30, 31, 34] with a total of 19 198 patients. One of these studies [34] did not report a standard deviation (SD), which rendered its data non-estimable for pooling in the meta-analysis. Consequently, only six studies were quantitatively analyzed. Statistical analysis demonstrated a significantly shorter operative time in patients with a BMI < 30 kg/m^2^ (SMD − 0.15; 95% CI: − 0.28 to − 0.02; *p* = 0.02) (Fig. 5C). The level of heterogeneity was substantial (I² = 75%; Chi² test: *p* = 0.001) and the level of certainty of evidence based on the GRADE criteria was very low (Tab. S7). Due to this substantial heterogeneity, a leave-one-out sensitivity analysis was performed, which identified the study by Özdemir et al. [27] as the primary source of heterogeneity. After excluding this study, heterogeneity decreased (I² = 58%; Chi² test: *p* = 0.05), and the pooled results were recalculated using a random-effects model (SMD − 0.20; 95% CI: − 0.31 to − 0.10; *p* = 0.0001) (Fig. 5D).

#### Postoperative bleeding

##### BMI < 25 kg/m^2^ versus BMI ≥ 25 kg/m^2^

Postoperative bleeding was reported in six studies [14, 27, 28, 31–33] with a total of 18 539 patients. Statistical analysis showed a significantly lower risk of bleeding in patients with a BMI < 25 kg/m^2^ compared to those with a BMI ≥ 25 kg/m^2^ (OR 0.74; 95% CI: 0.57–0.97; *p* = 0.03) (Fig. 5E). The level of heterogeneity was low (I² = 0%; Chi² test: *p* = 0.53). The quality of evidence was very low according to the GRADE criteria (Tab. S6).

##### BMI < 30 kg/m^2^ versus BMI ≥ 30 kg/m^2^

Postoperative bleeding was reported in seven studies [14, 16, 27, 30–33] with a total of 19 377 patients. Statistical analysis revealed no significant difference in bleeding risk between patients with BMI < 30 and ≥ 30 (OR 0.85; 95% CI: 0.69–1.05; *p* = 0.14) (Fig. 5F). The level of heterogeneity was low (I² = 0%; Chi² test: *p* = 0.93).

#### Length of hospital stay

##### BMI < 25 kg/m^2^ versus BMI ≥ 25 kg/m^2^

The Length of hospital stay was reported in four studies [14, 27, 28, 31] with a total of 16 835 patients. Two of these studies [14, 31] reported a standard deviation (SD) of zero, which rendered their data non-estimable for pooling in the meta-analysis. Consequently, only two studies [27, 28] were quantitatively analyzed. Statistical analysis revealed no significant difference in the length of stay between patients with a BMI < 25 kg/m^2^ and ≥ 25 kg/m^2^ (SMD 0.09; 95% CI: − 0.09 to 0.26; *p* = 0.33) (Fig. S1A). The level of heterogeneity was low (I² = 0%; Chi² test: *p* = 0.44).

##### BMI < 30 kg/m^2^ versus BMI ≥ 30 kg/m^2^

Length of hospital stay was reported in six studies [14, 16, 27, 30, 31, 34] with a total of 17 888 patients. Three of these studies [14, 31, 34] reported a standard deviation (SD) of zero or no SD, rendering their data non-estimable for pooling in the meta-analysis. Consequently, only three studies [16, 27, 30] were quantitatively analyzed. Statistical analysis revealed no significant difference in length of stay between patients with a BMI < 30 kg/m^2^ and ≥ 30 kg/m^2^ (SMD 0.35; 95% CI: − 0.39 to 1.08; *p* = 0.35) (Fig. S1B). The level of heterogeneity was considerable (I² = 97%; Chi² test: *p* < 0.00001). Due to this heterogeneity, a leave-one-out sensitivity analysis was performed, which identified the study by Vaghiri et al. [16] as the primary source of heterogeneity. After excluding this study, heterogeneity markedly decreased (I² = 19%; Chi² test: *p* = 0.27), and the pooled results were recalculated using a fixed-effects model (SMD − 0.07; 95% CI: − 0.21 to 0.06; *p* = 0.30) (Fig. S1C).

#### Surgical site infection

##### BMI < 25 kg/m^2^ versus BMI ≥ 25 kg/m^2^

Surgical site infection (SSI) was reported in three studies [15, 32, 33] including a total of 2 943 patients. Statistical analysis revealed no significant difference in SSI incidence between patients with BMI < 25 and ≥ 25 (OR 0.57; 95% CI: 0.22–1.45; *p* = 0.24) (Fig. S1D). The level of heterogeneity was low (I² = 0%; Chi² test: *p* = 0.93).

##### BMI < 30 kg/m^2^ versus BMI ≥ 30 kg/m^2^

Surgical site infection (SSI) was reported in five studies [15, 16, 30, 32, 33] with a total of 4 047 patients. Statistical analysis revealed no significant difference in SSI incidence between patients with BMI < 30 kg/m^2^ and ≥ 30 kg/m^2^ (OR 0.90; 95% CI: 0.28–2.91; *p* = 0.87) (Fig. S1E). The level of heterogeneity was low (I² = 0%; Chi² test: *p* = 0.97).

## Discussion

With the global prevalence of obesity continuing to rise, its impact on surgical practice is becoming increasingly significant, as surgeons encounter an ever-growing proportion of patient with overweight and obesity in routine clinical care [5]. Despite robust evidence linking obesity to adverse outcomes in other surgical fields, its influence on operative results following thyroid surgery remains insufficiently characterized [7–11]. This gap in knowledge is of considerable clinical relevance, given that thyroidectomy is a commonly performed surgical procedures [35, 36]. Therefore, the central objective of this meta-analysis was to synthesize the current evidence to systematically evaluate the safety of total thyroidectomy in individuals with overweight and obesity.

Defining appropriate eligibility criteria presented a significant challenge due to considerable inconsistencies in outcome reporting across the available literature and heterogenous definitions of operative complications among studies. To minimize variability, we included only studies reporting outcomes of conventional open total thyroidectomy with data stratified by BMI categories.

Our analysis of the data from the 12 studies included in our meta-analysis suggests that total thyroidectomy can be performed safely in patients with an elevated BMI with respect to recurrent laryngeal nerve palsy and permanent hypoparathyroidism, as no significant association between BMI and either complication was observed. Interestingly, postoperative and transient hypoparathyroidism occurred less frequently in patients with elevated BMI; however, this association did not extend to permanent hypoparathyroidism, which represents the clinically most relevant form of postoperative parathyroid dysfunction because of its long-term consequences for affected patients. Among the secondary perioperative outcomes, higher BMI was consistently associated with prolonged operative time. The findings also suggest a possible increased risk of postoperative hemorrhage, although this association was not consistent across both BMI cut-offs. No BMI-dependent differences were observed in surgical site infections or length of hospital stay.

Eight of the twelve studies included in our meta-analysis reported the incidence of postoperative RLN palsy, and only five of these differentiated between transient and permanent lesions. Based on the available data, we found no significant association between postoperative RLN palsy and patients’ BMI. The only meta-analysis published to date examining the association between BMI and complication rates in thyroid surgery interestingly reached a different conclusion. In their analysis, Gahni et al. reported an increased risk of postoperative recurrent laryngeal nerve injury among patients with obesity [37]. However, that meta-analysis also included studies reporting outcomes from both hemithyroidectomy and total thyroidectomy, making it impossible to determine whether the number of nerves at risk during surgery was truly comparable across BMI groups. From our perspective, further research is warranted to clarify whether obesity independently contributes to the risk of recurrent laryngeal nerve palsy.

Hypoparathyroidism caused by inadvertent injury, devascularization, or removal of the parathyroid glands remains one of the most frequent and clinically significant complications following thyroidectomy [38, 39]. Based on the results of the studies included in this meta-analysis, patients with elevated BMI were less likely to develop postoperative hypoparathyroidism. However, when outcomes were stratified into transient and permanent hypoparathyroidism, this association was confined to transient hypoparathyroidism, whereas no significant association was observed for permanent hypoparathyroidism. Several authors have reported the phenomenon that patients with obesity are less likely to develop postoperative hypoparathyroidism after thyroid surgery than individuals without obesity [14, 29, 40], a finding often referred to as the “obesity paradox.” Proposed explanations include a potential protective effect of increased cervical adipose tissue, which may shield the parathyroid glands from injury during surgical dissection. Another explanatory hypothesis centers on the chronically elevated parathyroid hormone secretion often observed in individuals with overweight and obesity [41], which may enhance their compensatory capacity to counteract postoperative hypoparathyroidism [14, 40]. Importantly, however, no significant association was observed between BMI and permanent hypoparathyroidism, which represents the clinically most relevant form of postoperative parathyroid dysfunction because of its long-term consequences. Thus, while the lower incidence of transient hypoparathyroidism in patients with elevated BMI is an interesting finding, our results do not indicate a BMI-dependent difference in the risk of permanent hypoparathyroidism following total thyroidectomy.

Postoperative hemorrhage following thyroid surgery is an uncommon but potentially life-threatening complication [42]. In our analysis, patients with overweight exhibited a significantly increased risk of postoperative bleeding compared with normal-weight individuals. However, this association was not confirmed when comparing patients with obesity with patients without obesity. In their meta-analysis, Ghani et al. reported an increased risk of postoperative hemorrhage after thyroid surgery among patients with obesity. Taken together, the available evidence suggests a possible association between elevated BMI and postoperative bleeding. However, the lack of consistent findings across both BMI cut-offs in our analysis warrants cautious interpretation. This potential association may, in part, reflect anatomical features commonly seen in individuals with obesity, such as a short, broad neck and reduced cervical extension, that can increase the technical complexity of thyroidectomy [43, 44]. As potential strategies to reduce the risk of postoperative hemorrhage in patients with a high BMI, authors have proposed meticulous attention to patient positioning and the use of larger incisions to improve surgical exposure, as well as increasing the application of energy devices [37, 42].

The previously described surgical challenges associated with thyroid procedures in patients with obesity may also account for the significantly longer operative times we observed among individuals with overweight and obesity. compared with patients with normal weight or without obesity.

In our analysis, length of hospital stay did not differ significantly across BMI categories. The same was true for postoperative wound infections, for which no BMI-related variation was observed. Overall, surgical site infections after thyroidectomy are uncommon, with reported incidence rates generally below 1% [45, 46]. Thyroidectomy is classified as a clean procedure, as neither the aerodigestive nor the gastrointestinal mucosa is entered and the wound is closed primarily [47]. Favorable wound healing in the cervical region may also be supported by the excellent perfusion of the neck’s soft tissues, taken together, these factors may help explain why BMI does not appear to influence the risk of wound infection in the context of thyroidectomy.

As with any meta-analysis, several limitations warrant consideration. Ten of the twelve included studies were retrospective, many from single centers, inherently introducing the risk of unmeasured confounding and both information and selection biases. Marked heterogeneity was evident in operative time and length of hospital stay, likely reflecting differences in the frequency of concomitant neck dissections as well as variability in surgeon experience, institutional volume, and patient comorbidity profiles. Regarding postoperative hemorrhage, the absence of reliable data on patients’ use of anticoagulant medications is a noteworthy limitation, and most studies did not distinguish whether bleeding events required surgical re-exploration. Further constraints include inconsistent definitions of postoperative hypoparathyroidism, variability in the use of intraoperative nerve monitoring, and differing postoperative diagnostic approaches for identifying RLN palsy. An additional source of potential confounding is the heterogeneous inclusion of patients with benign and malignant thyroid disease. Patients with thyroid carcinoma may undergo concomitant lymph node dissection, which is associated with an increased risk of postoperative hypoparathyroidism and recurrent laryngeal nerve injury. However, the included studies inconsistently reported the indication for surgery, the extent of lymph node dissection, and outcomes according to surgical extent. Consequently, the independent influence of these factors could not be evaluated. A further limitation relates to the use of BMI as the primary anthropometric measure. Although BMI is widely used and internationally accepted for the classification of overweight and obesity, it does not adequately reflect body composition or fat distribution. Alternative anthropometric parameters, such as waist-to-hip ratio and neck circumference, may provide a more comprehensive assessment of body fat distribution. In thyroid surgery, neck circumference may be particularly relevant, as it may better reflect the amount of cervical adipose tissue than BMI. Increased cervical adiposity may impair surgical exposure, hinder anatomical dissection, and thereby increase the technical complexity of thyroidectomy. Accordingly, procedure-specific anthropometric measures such as neck circumference might be more closely associated with perioperative outcomes following thyroidectomy than BMI alone. However, these parameters were not reported in the included studies and therefore could not be evaluated in the present meta-analysis. Finally, the methods used to determine BMI were inconsistently reported across the included studies. While several studies described preoperative measurements of height and weight, and one study additionally assessed body composition using bioelectrical impedance analysis (BIA), most studies did not specify how BMI was determined. Consequently, potential measurement bias related to BMI assessment cannot be excluded and should be considered when interpreting the findings of the present meta-analysis.

## Conclusion

Thyroidectomy is generally a safe procedure with a low rate of complications. According to the findings of our meta-analysis, this also appears to apply to patients with overweight or obesity, as we observed no increased risk of recurrent laryngeal nerve palsy or permanent hypoparathyroidism in these patient groups. However, operative time was significantly longer in patients with elevated BMI. Furthermore, our findings indicate a possible increased risk of postoperative hemorrhage in patients with overweight, although this association was not confirmed in patients with obesity. Given these observations, particular attention to meticulous intraoperative hemostasis may be warranted in patients with elevated BMI. 

## Supplementary Information

Below is the link to the electronic supplementary material.

Supplementary Material 1

Supplementary Material 2

Supplementary Material 3

## Data Availability

No datasets were generated or analysed during the current study.

## Abbreviations

BMI: Body mass index
LOS: Length of stay
LND: Lymph node dissection
HPT: Postoperative Hypoparathyroidism
RLN: Recurrent laryngeal nerve
SSI: Surgical site infection
TT: Total thyroidectomy
