# Supplementary material for: Impact of BMI on perioperative outcomes following total thyroidectomy: a systematic review and meta-analysis
Source: Thyroid Res. 2026 Oct 14;19:31. doi: 10.1186/s13044-026-00318-2 (PMC13663210; doi:10.1186/s13044-026-00318-2)
Supplement: Supplementary file 2 [file 13044_2026_318_MOESM2_ESM.docx]

**Supplementary material:**

Table S1: Definitions of primary outcomes

| **Author** | **Cut-off for transient vs permanent** | **Hypoparathyroidism** | **Hypocalcaemia** | **RLN palsy** |
| --- | --- | --- | --- | --- |
| **Blanchard et al. (18)** | 6 months | NR | Albumin corrected serum Calcium <2.0mmol/L at post-op day 2 | Vocal cord dysfunction in laryngoscopy |
| **Canu et al. (33)** | 12 months | PTH levels <10pg/mL | NR | Vocal cord dysfunction in laryngoscopy |
| **Celik et al. (37)** | 6 months | NR | Albumin corrected serum calcium  levels of <8.0 mg/dL | NR |
| **Eminov et al. (34)** | 6 months | NR | Albumin corrected serum Calcium <8 mg/dL or need for Calcium substitution | Vocal cord paralysis in indirect laryngoscopy |
| **Feng et al. (36)** | NA | NA | Calcium level < 2 mmol/L | Vocal cord dysfunction in laryngoscopy |
| **Mahvi et al. (32)** | 30 days | NR | Albumin corrected serum Calcium <8.3 | NR |
| **Milone et al. (31)** | 6 months | NR | Albumin corrected serum Calcium <2 mmol/L | Vocal cord dysfunction in laryngoscopy |
| **Özdemir et al. (30)** | >6 months | PTH levels <15mg/L | Albumin corrected serum Calcium <8.0mg/dL on post-op day 2 | Vocal cord mobility dysfunction in indirect laryngoscopy |
| **Soelling et al. (17)** | NA | NR | NR | Voice hoarseness or vocal cord dysfunction in laryngoscopy |
| **Tresallet et al. (35)** | 6 months | Hypocalcaemia and low parathyroid hormone levels requiring calcium and a-calcidol supplementation | Calcium levels <2 mmol/L | Vocal cord dysfunction in laryngoscopy |
| **Üstün et al. (29)** | 6 months | NA | Albumin corrected serum Calcium <8 mg/dL | Vocal cord dysfunction in indirect laryngoscopy, which was performed on patients with dysphonia, dyspnea, and swallowing disorders |
| **Vaghiri et al. (19)** | > 6 months | PTH levels <1.6pmol/L + hypocalcemia (with or without symptoms) + need for Calcium and/or Vitamin D substitution | Albumin corrected serum Calcium <2.1 mmol/L | Voice hoarseness and vocal cord dysfunction in laryngoscopy |

NR: Not reported; PTH: Parathyroid hormone; RLN: Recurrent laryngeal nerve;

Table S2: Primary outcomes in patients with BMI <25 kg/m² vs ≥25 kg/m²

| **Author** | **Group** | **Number of patients (n)** | **Postop. HPT (n)** | **Transient HPT (n)** | **Permanent HPT (n)** | **Postop. RLN palsy (n)** | **Transient RLN palsy (n)** | **Permanent RLN palsy (n)** |
| --- | --- | --- | --- | --- | --- | --- | --- | --- |
| **Blanchard et al. (18)** | BMI <25 | 595 | 140 | 128 | 12 | 56 | 53 | 3 |
|  | BMI ≥25 | 715 | 124 | 110 | 14 | 73 | 65 | 8 |
| **Eminov et al. (34)** | BMI <25 | 25 | 5 | NR | NR | 4 | NR | NR |
|  | BMI ≥25 | 74 | 11 | NR | NR | 8 | NR | NR |
| **Feng et al. (36)** | BMI <25 | 260 | 45 | NR | NR | 13 | NR | NR |
|  | BMI ≥25 | 157 | 32 | NR | NR | 11 | NR | NR |
| **Mahvi et al. (32)** | BMI <25 | 168 | 55 | NR | NR | NR | NR | NR |
|  | BMI ≥25 | 390 | 91 | NR | NR | NR | NR | NR |
| **Milone et al. (31)** | BMI <25 | 162 | 6 | NR | 6 | 6 | 4 | 2 |
|  | BMI ≥25 | 104 | 2 | NR | 2 | 4 | 3 | 1 |
| **Özdemir et al. (30)** | BMI <25 | 81 | 8 | 8 | NR | 4 | 2 | 2 |
|  | BMI ≥25 | 334 | 15 | 15 | NR | 19 | 9 | 10 |
| **Soelling et al. (17)** | BMI <25 | 3531 | 363 | NR | NR | 237 | NA | NR |
|  | BMI ≥25 | 12595 | 1021 | NR | NR | 908 | NA | NR |
| **Tresallet et al. (35)** | BMI <25 | 684 | 172 | 146 | 26 | 18 | 13 | 5 |
|  | BMI ≥25 | 532 | 108 | 85 | 23 | 16 | 10 | 6 |
| **Üstün et al. (29)** | BMI <25 | 66 | 25 | 25 | 0 | 1 | 1 | 0 |
|  | BMI ≥25 | 79 | 25 | 23 | 2 | 3 | 3 | 0 |

NR: Not reported; HPT: Hypoparathyroidism; RLN: Recurrent laryngeal nerve;

Table S3: Primary outcomes in patients with BMI <30 kg/m² vs ≥30 kg/m²

| **Author** | **Group** | **Number of patients (n)** | **Postop. HPT (n)** | **Transient HPT (n)** | **Permanent HPT (n)** | **Postop. RLN palsy (n)** | **Transient RLN palsy (n)** | **Permanent RLN palsy (n)** |
| --- | --- | --- | --- | --- | --- | --- | --- | --- |
| **Blanchard et al. (18)** | BMI <30 | 1020 | 219 | 198 | 21 | 96 | 88 | 8 |
|  | BMI ≥30 | 290 | 45 | 40 | 5 | 33 | 30 | 3 |
| **Canu et. al. (33)** | BMI <30 | 678 | 184 | 147 | 37 | 22 | NR | NR |
|  | BMI ≥30 | 135 | 36 | 25 | 11 | 3 | NR | NR |
| **Celik et al. (37)** | BMI <30 | 66 | 15 | 15 | 0 | NR | NR | NR |
|  | BMI ≥30 | 78 | 13 | 13 | 0 | NR | NR | NR |
| **Eminov et al. (34)** | BMI <30 | 61 | 10 | 10 | NR | 8 | 8 | NR |
|  | BMI ≥30 | 38 | 6 | 6 | NR | 4 | 4 | NR |
| **Feng et al. (36)** | BMI <30 | 392 | 72 | NR | NR | 21 | NR | NR |
|  | BMI ≥30 | 25 | 5 | NR | NR | 3 | NR | NR |
| **Özdemir et al. (30)** | BMI <30 | 245 | 17 | 17 | NR | 17 | 7 | 10 |
|  | BMI ≥30 | 170 | 6 | 6 | NR | 6 | 4 | 2 |
| **Soelling et al. (17)** | BMI <30 | 8354 | 746 | NR | NR | 571 | NR | NR |
|  | BMI ≥30 | 7772 | 638 | NR | NR | 574 | NR | NR |
| **Tresallet et al. (35)** | BMI <30 | 1040 | 239 | 201 | 38 | 27 | 21 | 6 |
|  | BMI ≥30 | 176 | 41 | 30 | 11 | 7 | 2 | 5 |
| **Vaghiri et al. (19)** | BMI <30 | 193 | 78 | 56 | 22 | 33 | 27 | 6 |
|  | BMI ≥30 | 98 | 36 | 21 | 15 | 13 | 11 | 2 |

NR: Not reported; HPT: Hypoparathyroidism; RLN: Recurrent laryngeal nerve;

Table S4: Secondary outcomes in patients with BMI <25 kg/m² vs ≥25 kg/m²

| **Author** | **Group** | **Number of patients (n)** | **OR time in minutes (mean/SD)** | **LOS (mean /SD)** | **SSI (n)** | **Bleeding (n)** |
| --- | --- | --- | --- | --- | --- | --- |
| **Blanchard et al. (18)** | BMI <25 | 595 | 96.2 ± 39.8 | NR | 1 | NR |
|  | BMI ≥25 | 715 | 104.5 ± 43.2 | NR | 2 | NR |
| **Eminov et al. (34)** | BMI <25 | 25 | 33.6 ± 7.4 | 1 ± 0.2 | NR | 1 |
|  | BMI ≥25 | 74 | 38.9 ± 12.8 | 1 ± 0 | NR | 0 |
| **Feng et al. (36)** | BMI <25 | 260 | NR | NR | 5 | 2 |
|  | BMI ≥25 | 157 | NR | NR | 6 | 3 |
| **Mahvi et al. (32)** | BMI <25 | 168 | 125 ± 59 | NR | NR | NR |
|  | BMI ≥25 | 390 | 124 ± 51 | NR | NR | NR |
| **Milone et al. (31)** | BMI <25 | 162 | NR | 2.8 ± 0.6 | NR | 13 |
|  | BMI ≥25 | 104 | NR | 2.7 ± 0.7 | NR | 7 |
| **Özdemir et al. (30)** | BMI <25 | 81 | 84.1 ± 27.9 | 1.6 ±1.7 | NR | 0 |
|  | BMI ≥25 | 334 | 80.2 ± 25.2 | 1.4 ± 12.3 | NR | 3 |
| **Soelling et al. (17)** | BMI <25 | 3531 | 123 ± 57 | 1.3 ± 0.7 | NR | 53 |
|  | BMI ≥25 | 12595 | 130.1 ± 58 | 1 ± 0 | NR | 262 |
| **Tresallet et al. (35)** | BMI <25 | 684 | NR | NR | 2 | 4 |
|  | BMI ≥25 | 532 | NR | NR | 2 | 6 |
| **Üstün et al. (29)** | BMI <25 | 66 | 148.4 ± 0 | NR | NR | NR |
|  | BMI ≥25 | 79 | 153.4 ± 0 | NR | NR | NR |

OR: Operating room; SD: Standard deviation; LOS: Length of hospital stay; SSI: Surgical site infection;

Table S5: Secondary outcomes in patients with BMI <30 kg/m² vs ≥30 kg/m²

| **Author** | **Group** | **Number of patients (n)** | **OR time in minutes (mean/SD)** | **LOS (mean /SD)** | **SSI (n)** | **Bleeding (n)** |
| --- | --- | --- | --- | --- | --- | --- |
| **Blanchard et al. (18)** | BMI <30 | 1020 | 99.2 ± 40.9 | NR | 2 | NR |
|  | BMI ≥30 | 290 | 106.2 ± 44.8 | NR | 1 | NR |
| **Canu et. al. (33)** | BMI <30 | 678 | 86.6 ± 19.2 | 2.9 ± 1.6 | 2 | 9 |
|  | BMI ≥30 | 135 | 92.8 ± 20.8 | 3.2 ± 1.2 | 0 | 0 |
| **Celik et al. (37)** | BMI <30 | 66 | 118.5 | 1,3 | NR | NR |
|  | BMI ≥30 | 78 | 116.3 | 1,4 | NR | NR |
| **Eminov et al. (34)** | BMI <30 | 61 | 36.7 ± 10.5 | 1 ± 0.1 | NR | 1 |
|  | BMI ≥30 | 38 | 38.8 ± 13.3 | 1 ± 0 | NR | 0 |
| **Feng et al. (36)** | BMI <30 | 392 | NR | NR | 10 | 5 |
|  | BMI ≥30 | 25 | NR | NR | 1 | 0 |
| **Özdemir et al. (30)** | BMI <30 | 245 | 83.1 ± 26.3 | 1.5 ± 1.7 | NR | 2 |
|  | BMI ≥30 | 170 | 77.9 ± 25.1 | 1.4 ± 17 | NR | 1 |
| **Soelling et al. (17)** | BMI <30 | 8354 | 125.3 ± 57.6 | 1.1 ± 0.5 | NR | 150 |
|  | BMI ≥30 | 7772 | 132 ± 58 | 1 ± 0 | NR | 165 |
| **Tresallet et al. (35)** | BMI <30 | 1040 | NR | NR | 4 | 8 |
|  | BMI ≥30 | 176 | NR | NR | 0 | 2 |
| **Vaghiri et al. (19)** | BMI <30 | 193 | 191.3 ± 61.2 | 21.3 ± 13.8 | 1 | 6 |
|  | BMI ≥30 | 98 | 217.3 ± 73.3 | 7.5 ± 4 | 0 | 5 |

OR: Operating room; SD: Standard deviation; LOS: Length of hospital stay; SSI: Surgical site infection;

Table S6: GRADE Certainty assessment for significant outcomes (BMI <25 kg/m² vs BMI ≥25 kg/m²)

| Outcomes | No. of studies | No. of included patients | | OR/SMD [95 % CI] | Quality assessment | | | | | Quality |
| --- | --- | --- | --- | --- | --- | --- | --- | --- | --- | --- |
|  |  | BMI<25 | BMI ≥25 |  | Risk of bias^a^ | Inconsistency | Indirectness | Imprecision | Publication bias |  |
| Postoperative hypoparathyroidism | 9 [17,18,29–32,34–36] | 819/5572 (14.7%) | 1429/14980 (9.5%) | 1.33 [1.20 to 1.46] | Serious | Not serious | Not serious | Not serious | Undetected | Low |
| Transient hypoparathyroidism | 4 [18,29,30,35] | 307/1426 (21.5%) | 233/1660 (14.0%) | 1.50 [1.24 to 1.81] | Serious | Not serious | Not serious | Not serious | Undetected | Low |
| Postoperative Bleeding | 6 [17,30,31,34–36] | 73/4743 (1.5%) | 281/13796 (2.0%) | 0.74 [0.57 to 0.97] | Serious | Not serious | Not serious | Serious | Undetected | Very low |
| Operative time^b^ | 5 [17,18,30,32,34] | 4385 | 13783 | -0.13 [-0.22 to -0.04] | Serious | Serious | Not serious | Not serious | Undetected | Very low |
| OR: odds ratio; SMD: standardized mean difference; ^a^ Risk of bias assessed using the ROBINS-I tool; ^b^Operative time after excluding Özdemir et al. ^30^ | | | | | | | | | | |

Table S7: GRADE Certainty assessment for significant outcomes (BMI <30 kg/m² vs BMI ≥30 kg/m²)

| Outcomes | No. of studies | No. of included patients | | OR/SMD [95 % CI] | Quality assessment | | | | | Quality |
| --- | --- | --- | --- | --- | --- | --- | --- | --- | --- | --- |
|  |  | BMI<30 | BMI ≥30 |  | Risk of bias^a^ | Inconsistency | Indirectness | Imprecision | Publication bias |  |
| Postoperative hypoparathyroidism | 9 [17–19,30,33–37] | 1580/12049 (13.1%) | 826/8782 (9.4%) | 1.12 [1.02 to 1.24] | Serious | Not serious | Not serious | Not serious | Undetected | Low |
| Transient hypoparathyroidism | 7 [18,19,30,33–35,37] | 644/3303 (19.5%) | 141/985 (14.3%) | 1.37 [1.11 to 1.68] | Serious | Not serious | Not serious | Not serious | Undetected | Low |
| Operative time | 7 [17–19,30,33,34,37] | 10617 | 8581 | -0.15 [-0.28 to -0.02] | Serious | Serious | Not serious | Not serious | Undetected | Very Low |
| OR: odds ratio; SMD: standardized mean difference, ^a^ Risk of bias assessed using the ROBINS-I tool | | | | | | | | | | |

**Figure Legend**

Supplementary Figure 1. (A) Forest plot comparing length of hospital stay between patients with BMI <25 kg/m^2^ and ≥25 kg/m^2^. (B) Forest plot comparing length of hospital stay between patients with BMI <30 kg/m^2^ and ≥30 kg/m^2^. (C) Leave-one-out sensitivity analysis: Forest plot comparing length of hospital stay between patients with BMI <30 kg/m^2^ and ≥30 kg/m^2^. (D) Forest plot comparing surgical site infection between patients with BMI <25 kg/m^2^ and ≥25 kg/m^2^. (E) Forest plot comparing surgical site infection between patients with BMI <30 kg/m^2^ and ≥30 kg/m^2^.

**Search strategy**

The literature search was conducted using the search terms "BMI," "obesity," and "thyroidectomy." The search was performed across the following electronic databases: PubMed, Cochrane Library, and Google Scholar.
